# Soaking in Povidone-Iodine, Chlorhexidine, Teicoplanin, Vancomycin, and Saline Solution Differentially Alters Porcine Flexor Tendon Size and Biomechanical Properties

**DOI:** 10.1016/j.asmr.2025.101168

**Published:** 2025-05-21

**Authors:** Kaan Gurbuz, Yukun Zhang, Logan Opperman, Matthew B. Fisher

**Affiliations:** a Department of Orthopedics and Traumatology, Kayseri Medical Faculty, University of Health Sciences, Kayseri, Turkey; b Lampe Joint Department of Biomedical Engineering, North Carolina State University and University of North Carolina at Chapel Hill, Raleigh, North Carolina, U.S.A.; c Comparative Medicine Institute, North Carolina State University, Raleigh, North Carolina, U.S.A.; d Department of Statistics, North Carolina State University, Raleigh, North Carolina, U.S.A.

## Abstract

**Purpose:**

To compare the impact of povidone-iodine, chlorhexidine, vancomycin, and teicoplanin on the size and biomechanical properties of porcine flexor tendon grafts.

**Methods:**

Porcine deep digital flexor tendons (N = 120) were collected in pairs and allocated into povidone-iodine, chlorhexidine, vancomycin, teicoplanin, and saline solution groups (12 pairs per solution). The tendons from one side underwent a 30-minute soaking in these solutions, whereas those from the other side were wrapped in saline solution–soaked gauze. Tendon cross-sectional area (CSA) was measured before and after soaking, along with unsoaked controls. Tensile testing to failure was performed. Stiffness, load at failure, modulus, and stress at failure were calculated.

**Results:**

Soaking in povidone-iodine and chlorhexidine decreased the average CSA by 8% (–0.9 mm^2^ [95% confidence interval (CI), –1.7 to –0.2 mm^2^], *P* = .02) and 13% (–1.1 mm^2^ [95% CI, –1.5 to –0.7 mm^2^], *P* < .001), respectively. Conversely, the average CSA increased by 12% after soaking in teicoplanin (1.0 mm^2^ [95% CI, 0.7 to 1.3 mm^2^], *P* < .001) and by 6% after soaking in vancomycin (0.7 mm^2^ [95% CI, 0.2 to 1.2 mm^2^], *P* = .01), similar to saline solution (11% increase; 0.8 mm^2^ [95% CI, 0.4 to 1.1 mm^2^], *P* < .001). The stiffness of tendons soaked in chlorhexidine was 8% lower than that of unsoaked contralateral controls (–13.3 N/mm [95% CI, –31.0 to –6.9 N/mm], *P* = .01), but stress at failure increased by 15% (8.9 MPa [95% CI, 0.6 to 17.2 MPa], *P* = .04). We observed no significant difference in tensile properties due to soaking in other solutions.

**Conclusions:**

Soaking porcine flexor tendons in povidone-iodine and chlorhexidine resulted in a decrease in CSA, whereas soaking in vancomycin and teicoplanin led to an increase, similar to saline solution. No significant side-to-side differences in average CSA were observed. Soaking in chlorhexidine decreased stiffness but increased stress at failure. None of the solutions affected load at failure.

**Clinical Relevance:**

It is important to understand the impact of soaking porcine flexor tendon grafts in various antimicrobial solutions to ensure that there are no harmful biomechanical effects to the tissues used in the clinical setting.

Ligament reconstruction is a surgical procedure to restore the function and stability of a joint after injury and is commonly performed in the elbow, shoulder, and knee joints.^1^, ^2^, ^3^ Graft harvesting and preparation are required before implantation.^4^ One intraoperative challenge is graft contamination, which can occur through dropping or touching nonsterile objects.^3^^,^^4^ For example, during anterior cruciate ligament (ACL) reconstruction, graft contamination can be a cause of postoperative septic arthritis, potentially leading to cartilage loss and graft failure.^5^ Although instances of this situation are rare, with reported incidence rates up to 3.8%,^4^, ^5^, ^6^ surgeons must have a plan to eliminate intraoperative graft contamination to avoid these potential effects. Current options include cleansing or sterilizing the graft, harvesting a new graft, and delaying surgery.^4^

To minimize time, cost, and patient morbidity, soaking grafts in an antimicrobial solution is a common option to treat intraoperative graft contamination^4^^,^^7^ because it avoids the need for harvesting a new graft or delaying surgery.^4^ It is important to consider the effects of antimicrobial solutions on the biomechanical properties of grafts to avoid potential graft failure. Biomechanical studies showed no difference in the Young modulus and ultimate tensile stress in human semitendinosus tendons soaked in 5-mg/mL vancomycin versus normal saline solution for 10 to 15 minutes.^6^^,^^8^ Consistent with these findings, no significant changes in the Young modulus were observed in a separate study when soaking bovine patellar tendons in vancomycin solution for 30 minutes.^9^ Soaking human patellar tendon allografts in 4% chlorhexidine for 30 minutes showed similar failure load and stiffness to soaking in normal saline solution.^7^ Additionally, an in vivo rat ACL reconstruction study reported no adverse effects from chlorhexidine soaking at both 4- and 12-week follow-up assessments.^10^ However, the results of a direct comparison of the effects of soaking in povidone-iodine, chlorhexidine, vancomycin, teicoplanin, and saline solution on graft tendon biomechanics remain unknown. Additionally, previous work measured only the width and thickness of the tendons rather than the cross-sectional area (CSA),^6^^,^^7^ which did not allow tracking of the size changes before and after soaking. Comparison between unpaired specimens in previous work also introduced interindividual variability.^6^^,^^7^

The purpose of this study was to compare the impact of povidone-iodine, chlorhexidine, vancomycin, and teicoplanin on the size and biomechanical properties of porcine flexor tendon grafts. We hypothesized that soaking tendon grafts in any antimicrobial solution would not change the size or biomechanical properties of the tendons.

## Methods

A total of 120 deep digital flexor tendons were collected from both hindlimbs of 60 female Yorkshire crossbreed pigs aged 1.5 months (juvenile) after humane euthanasia. The animals were bred at the North Carolina State University Swine Educational Unit, and animal use was approved by the North Carolina State University Institutional Animal Use and Care Committee. Subsequently, the specimens were wrapped in phosphate-buffered saline solution (PBS)–soaked gauze and stored at –20°C. Prior to testing, the specimens were thawed at room temperature (70°F).

**Fig 1:**
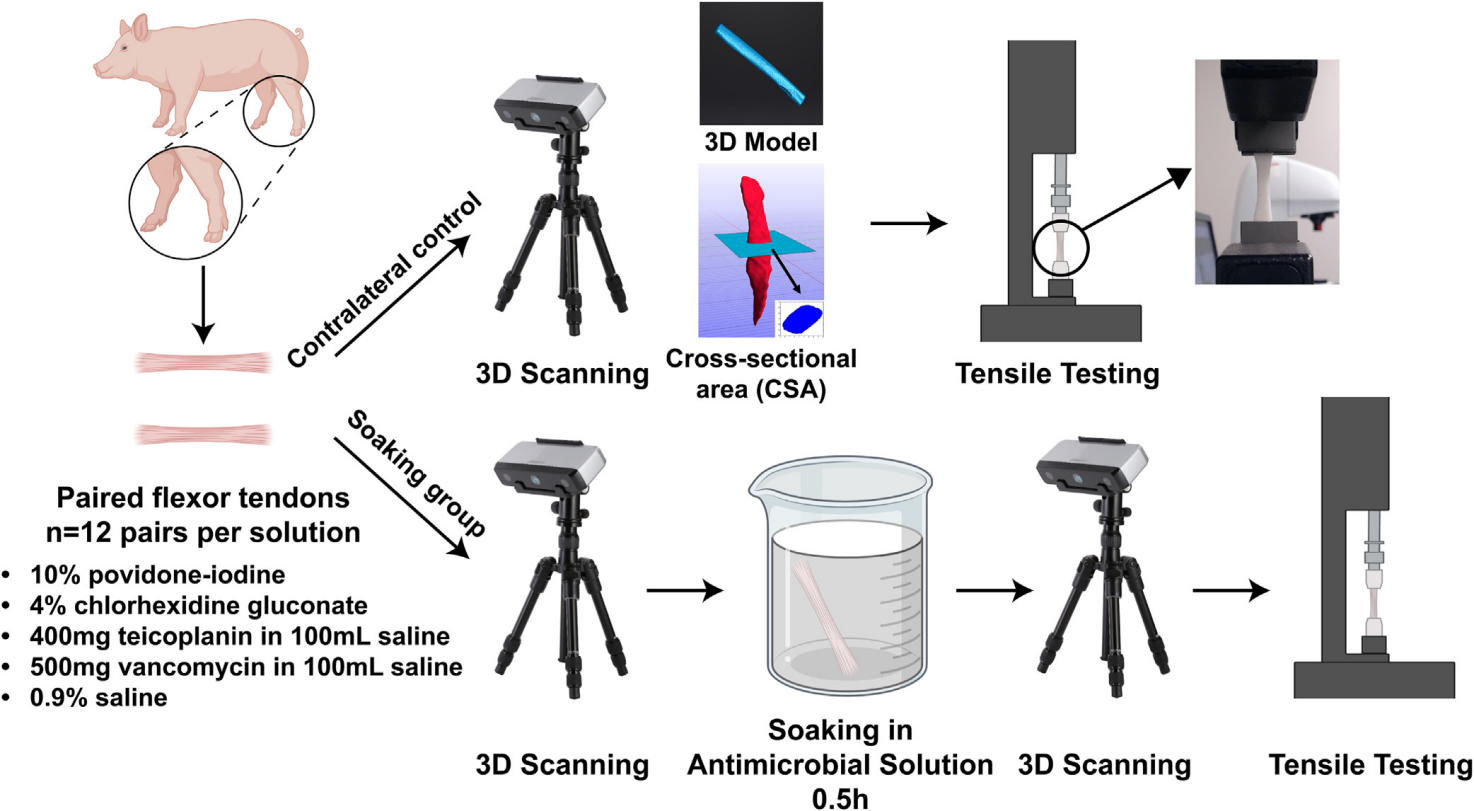
Experimental protocol overview. Deep digital flexor tendons were collected from both hindlimbs of juvenile pigs (aged 1.5 months). One side was used as the contralateral control whereas the other side was soaked in different antimicrobial solutions for 30 minutes. We performed 3-dimensional (3D) scanning of the contralateral controls and the tendons in the soaking groups before and after soaking, followed by tensile testing until failure.

The specimens were randomly divided into 5 cohorts in pairs. One side of the paired tendons was used as a contralateral control, whereas the other side was soaked in different antimicrobial solutions: 10% povidone-iodine solution; 4% chlorhexidine gluconate solution; 400 mg of teicoplanin in 100 mL of 0.9% saline solution; 500 mg of vancomycin in 100 mL of 0.9% saline solution; and 0.9% saline solution (12 pairs per solution). The concentrations were selected based on common use in the operating room and prior work.^4^^,^^8^^,^^11^ The tendons from one side underwent a 30-minute soaking in these solutions, whereas those from the contralateral control side were wrapped in gauze soaked in PBS. A 3-dimensional (3D) scanner (EinScan-SP; Shining 3D, Hangzhou, China) was used to measure the CSA of each tendon to allow a comparison of size as well as further analysis of stress (Appendix Fig 1). Three-dimensional scanning was performed before and after tendon soaking, along with scanning of the unsoaked contralateral control, to obtain a 3D model of each specimen. The CSA of the middle region was visualized in MATLAB (The MathWorks, Natick, MA), and the CSA value was calculated as the average CSA of the middle 50% of each 3D tendon model using custom MATLAB codes (Fig 1).

After 3D scanning, biomechanical testing of all specimens was performed on a universal testing system with a 2-kN load cell (Instron 5944; Instron, Norwood, MA) (Fig 1). Pneumatic clamps (6 bar) were used to avoid the potential sliding of specimens within the clamps (Appendix Fig 2). Tissue marking dye (Electron Microscopy Sciences, Hatfield, PA) was applied near the clamping area to monitor any sliding of the tendons. A 5-N preload was applied to the tendons, followed by cyclical preconditioning between 0% and 3% grip-to-grip strain at a constant rate of 0.1 mm/s. After 10 cycles, tensile extension was applied at a rate of 0.1 mm/s until specimen failure occurred.^12^^,^^13^ This relatively slow rate allowed for a smooth loading profile. Force and displacement were recorded. Stress was defined by force per CSA. Strain was defined by the percentage change in elongation relative to the original grip-to-grip length. The stiffness of each sample was calculated as the slope of the linear regression within the linear region of the force-displacement curve using MATLAB, with the linear region manually identified between the transition point following the toe region and the yield point. The modulus was calculated in a similar way from the stress-strain curve. Load at failure and stress at failure were taken at the point just before tendon rupture. Both the CSA measurements and tensile testing were performed by the same graduate student researcher (Y.Z.) with more than 5 years of experience in biomechanical testing. The researcher was not blinded to group allocation.

Statistical analysis was conducted using Prism (GraphPad Software, San Diego, CA). A paired *t* test was performed to assess the difference in CSA between the paired tendons before soaking, as well as for the same tendons before and after soaking. Tensile properties of the contralateral control tendons and the tendons after soaking, including stiffness, load at failure, modulus, and stress at failure, were also compared by a paired *t* test. The level of statistical significance was set at .05.

## Results

**Fig 2:**
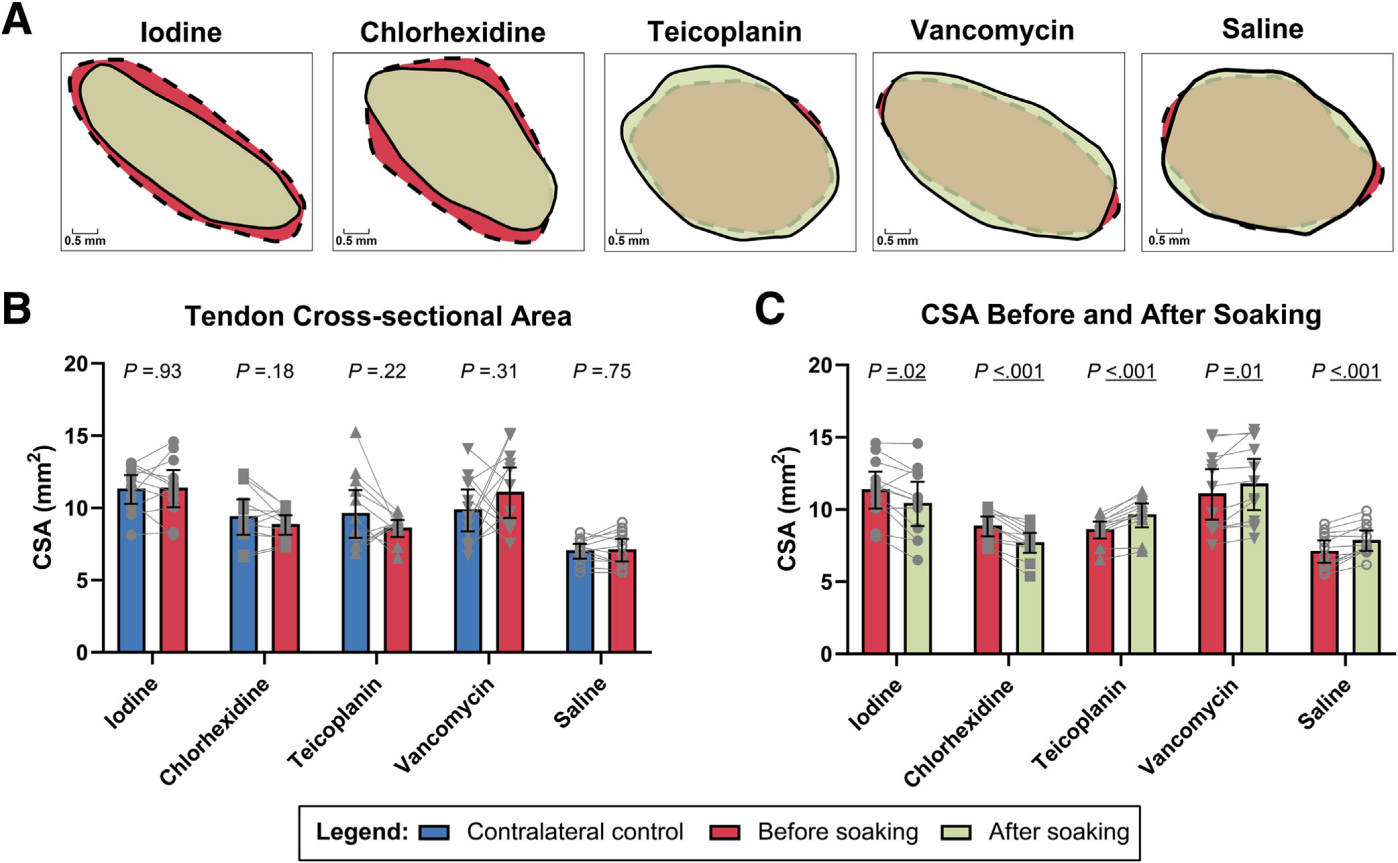
Soaking tendons in povidone-iodine solution and chlorhexidine solution led to a reduction in cross-sectional area (CSA), whereas soaking tendons in teicoplanin solution, vancomycin solution, and normal saline solution resulted in an increase in CSA. (A) Visualization of CSA of representative tendon samples before and after soaking in each solution. (B) There was no side-to-side difference in average CSA between contralateral controls and samples before soaking, but variation was observed in some of the animals. (C) On assessment of the same tendon, the change in CSA varied when soaking in different solutions. Mean values are shown as bars with 95% confidence intervals and paired samples connected. Statistical results are presented in the graphs.

The CSA of the middle region before and after soaking was observed from representative tendons in each solution (Fig 2A). For all samples, there was no significant difference in the average CSA between the tendons before soaking and the unsoaked contralateral tendons (*P* > .05 for all), with some pairs exhibiting side-to-side variation (Fig 2B, Table 1). The variability in CSA was compared within the same tendons before and after soaking (Fig 2C, Table 1). The average CSA decreased by 8% after soaking in povidone-iodine (–0.9 mm^2^ [95% confidence interval (CI), –1.7 to –0.2 mm^2^], *P* = .02) and decreased by 13% after soaking in chlorhexidine (–1.1 mm^2^ [95% CI, –1.5 to –0.7 mm^2^], *P* < .001). Conversely, the average CSA increased by 12% after soaking in teicoplanin (1.0 mm^2^ [95% CI, 0.7 to 1.3 mm^2^], *P* < .001), 6% after soaking in vancomycin (0.7 mm^2^ [95% CI, 0.2 to 1.2 mm^2^], *P* = .01), and 11% after soaking in normal saline solution (0.8 mm^2^ [95% CI, 0.4 to 1.1 mm^2^], *P* < .001).

**Table 1:** Cross-Sectional Area Comparison: Side-to-Side Differences and Differences Before Versus After Soaking

Solution	Cross-Sectional Area, mm^2^			*P* Value	
	Contralateral	Before Soaking	After Soaking	Contralateral vs Before Soaking (Side-to-Side Comparison)	Before Soaking vs After Soaking (Same Tendon)
Iodine	11.4 ± 1.6	11.4 ± 2.0	10.5 ± 2.4	.93	.02
Chlorhexidine	9.4 ± 2.0	8.9 ± 1.1	7.8 ± 1.1	.18	<.001
Teicoplanin	9.7 ± 2.6	8.7 ± 0.9	9.7 ± 1.3	.22	<.001
Vancomycin	9.9 ± 2.3	11.1 ± 2.8	11.8 ± 2.8	.31	.01
Saline	7.1 ± 0.8	7.2 ± 1.2	7.9 ± 1.1	.75	<.001

NOTE. Data are presented as mean ± standard deviation.

**Fig 3:**
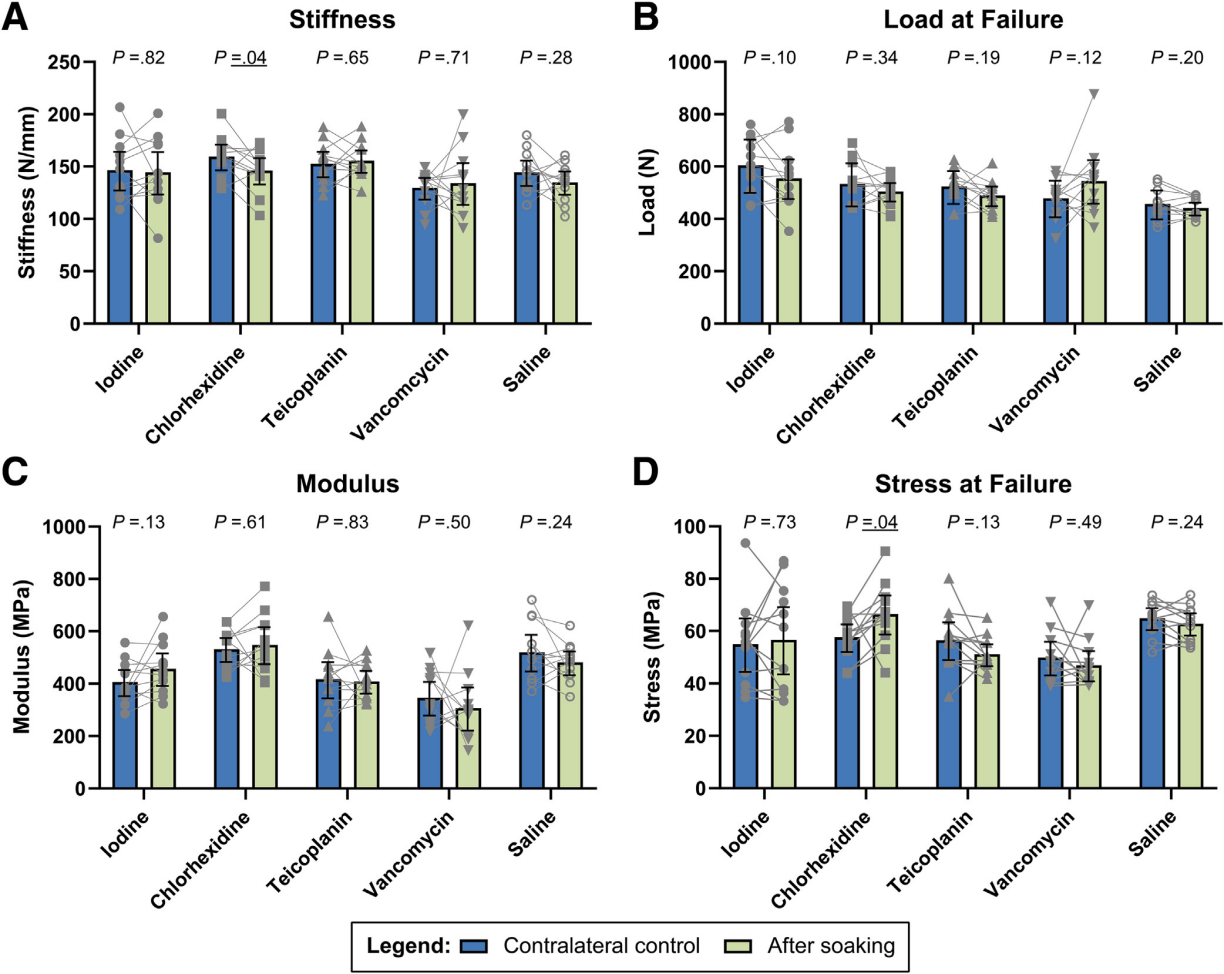
Differences in stiffness (A), load at failure (B), modulus (C), and stress at failure (D) between contralateral controls and soaked samples were quantified for povidone-iodine, chlorhexidine, teicoplanin, vancomycin, and normal saline solution. After soaking in chlorhexidine, stiffness was lower whereas stress at failure was higher compared with the contralateral control. Mean values are shown as bars with 95% confidence intervals and paired samples connected. Statistical results are presented in the graphs.

The average stiffness values of samples soaked in chlorhexidine were 8% smaller than those of the unsoaked contralateral controls (–13.3 N/mm [95% CI, –31.0 to –6.9 N/mm], *P* = .01) (Fig 3A, Table 2). There was no significant difference in stiffness for the other solution groups (*P* > .05 for each) (Fig 3A). The average load at failure and modulus were similar between the soaked samples and the contralateral controls (*P* > .05 for all) (Fig 3 B and C, Tables 2 and 3). The average stress at failure of the samples soaked in chlorhexidine was 15% greater than that of the contralateral controls (8.9 MPa [95% CI, 0.6 to 17.2 MPa], *P* = .04), whereas no significant differences were found for the other groups relative to the unsoaked contralateral controls (*P* > .05 for each) (Fig 3D, Table 3). Almost all samples failed near the clamp sites.

**Table 2:** Stiffness and Load at Failure: Contralateral Control Versus Soaking

Solution	Stiffness			Load at Failure		
	Contralateral, N/mm	Soaking, N/mm	*P* Value	Contralateral, N	Soaking, N	*P* Value
Iodine	146.5 ± 29.0	144.4 ± 32.0	.82	604.3 ± 102.1	554.9 ± 118.8	.10
Chlorhexidine	159.5 ± 19.2	146.2 ± 19.8	.01	533.3 ± 82.0	505.0 ± 55.7	.34
Teicoplanin	152.7 ± 19.2	155.6 ± 16.7	.65	523.3 ± 63.2	489.4 ± 58.7	.19
Vancomycin	129.7 ± 16.3	134.2 ± 31.3	.71	478.8 ± 69.6	544.7 ± 131.4	.12
Saline	144.5 ± 19.2	135.0 ± 17.5	.28	456.8 ± 55.7	441.0 ± 39.0	.20

NOTE. Data are presented as mean ± standard deviation.

**Table 3:** Modulus and Stress at Failure: Contralateral Control Versus Soaking

Solution	Modulus			Stress at Failure		
	Contralateral, MPa	Soaking, MPa	*P* Value	Contralateral, MPa	Soaking, MPa	*P* Value
Iodine	406.5 ± 78.5	457.0 ± 97.1	.13	55.0 ± 16.0	56.6 ± 20.3	.73
Chlorhexidine	531.4 ± 71.4	548.3 ± 111.1	.61	57.6 ± 8.3	66.5 ± 11.8	.04
Teicoplanin	416.8 ± 108.3	409.1 ± 68.3	.83	56.5 ± 11.3	51.1 ± 6.5	.13
Vancomycin	346.0 ± 101.2	306.9 ± 129.8	.50	49.8 ± 10.0	46.9 ± 9.1	.49
Saline	519.8 ± 109.8	481.0 ± 71.1	.24	64.9 ± 6.6	62.8 ± 6.3	.24

NOTE. Data are presented as mean ± standard deviation.

## Discussion

In this study, we found that although all 4 solutions differentially affected tendon size, only chlorhexidine significantly altered tensile properties. Soaking in povidone-iodine and chlorhexidine resulted in a decrease in CSA, whereas soaking in vancomycin and teicoplanin led to an increase. No significant changes in biomechanical properties, including stiffness, load at failure, modulus, and stress at failure, were observed between unsoaked contralateral control samples and tendons soaked in povidone-iodine, vancomycin, or teicoplanin. However, samples soaked in chlorhexidine exhibited lower average stiffness values than unsoaked contralateral controls, and stress at failure was higher. Although these differences may not necessarily translate into clinically significant effects that alter patient treatment decisions, they suggest minor—but detectable—biomechanical implications. It is important to note that, because almost all samples failed near the clamp sites, the stress at failure reported in this study may not reflect typical ultimate strength due to midsubstance tissue failure.

Although the cross-sectional shape of tendons is irregular, earlier studies measured tendon size using a digital caliper to assess width and thickness, potentially leading to size misestimation.^6^^,^^7^ Our study used a 3D scanner for a more comprehensive assessment of tendon size. Although no significant difference in average CSA was observed between tendons before soaking and unsoaked contralateral controls, side-to-side CSA variation was noted for some pairs, underscoring the importance of tracking individual tendon CSA before and after treatment. Previous comparisons of graft size were made between tendons soaked in antimicrobial solutions and those soaked in saline solution,^6^^,^^7^^,^^9^ whereas our study compared the CSA of the soaked sample with the CSA of the same sample prior to soaking, which could reduce interindividual variability. The changes in CSA after soaking in various solutions may be attributed to differences in osmolarity. However, the osmolarity of the solutions was not measured.

Chlorhexidine and povidone-iodine are antiseptic options for preventing bacterial growth during decontamination. An animal study showed that soaking rabbit patellar tendons contaminated with staphylococci in 4% chlorhexidine gluconate solution effectively decontaminated the tendons whereas 10% povidone-iodine solution was ineffective.^11^ Similarly, human data suggest that chlorhexidine provides more reliable disinfection than povidone-iodine.^14^^,^^15^ Moreover, a case series showed no postoperative infections after cleansing tendon grafts in chlorhexidine.^4^ However, although earlier research indicated that soaking human patellar tendon allografts in chlorhexidine had a negligible effect on structural properties,^7^ our results showed decreased stiffness in tendons soaked in chlorhexidine. The reduction in stiffness suggests a potential weakening of the tendon’s load-bearing capacity. This discrepancy may be attributed to differences in graft processing. In the previous work, grafts underwent standard irradiation prior to washing protocols,^7^ which is not feasible during intraoperative contamination management. Our samples were freshly frozen with PBS-soaked gauze prior to testing.

Soaking in vancomycin and teicoplanin, on the other hand, resulted in increased CSA and maintained comparable biomechanical properties. An animal study showed that soaking porcine flexor tendons in vancomycin effectively eliminated *Staphylococcus epidermidis* contamination when using concentrations of 5 or 10 mg/mL for at least 20 minutes.^16^ Case series and review studies have suggested that presoaking grafts in vancomycin significantly reduces the risk of postoperative knee infection after ACL reconstruction.^17^, ^18^, ^19^, ^20^, ^21^ A systematic review showed that vancomycin graft presoaking did not compromise the graft failure rate.^22^ Teicoplanin has been reported to cause fewer treatment discontinuations due to adverse events and to be less nephrotoxic compared with vancomycin when administered systemically.^23^, ^24^, ^25^ However, teicoplanin treatment is more expensive because of higher acquisition costs^26^, ^27^, ^28^ and is not typically available in the United States because it is not approved by the U.S. Food and Drug Administration.^29^ Therefore, the current recommendation for intraoperative graft cleansing is to soak the graft in vancomycin for as long as possible, given its minimal effects on tendon size and biomechanical properties, lower cost and higher availability compared with teicoplanin, and supporting evidence from clinical trials. Analyzing the impact of various antimicrobial solutions used for cleansing grafts can provide valuable insights to surgeons considering options for graft decontamination, which may allow for promising graft biomechanics before surgical procedures.

### Limitations

This study has some limitations. First, although a loading rate of 0.1 mm/s was chosen to align with prior biomechanical studies, this slow rate may not accurately replicate clinical traumatic failure or physiological loading conditions. Second, markers were not placed on the tendon to track regional modulus, so the strain measurements presented are based solely on clamp-to-clamp measurements. Third, the experimental conditions differed from the clinical scenario because this study used thawed animal tendons with a lack of biological healing. Furthermore, the study was unable to biomechanically test the same tendons as those used for CSA measurements, relying instead on contralateral controls, which may introduce side-to-side variability. No a priori sample size analysis was performed, so the study may be underpowered. Additionally, the comparisons in this work were limited to paired tendons without direct comparisons between different antimicrobial solutions.

## Conclusions

Soaking porcine flexor tendons in povidone-iodine and chlorhexidine resulted in a decrease in CSA, whereas soaking in vancomycin and teicoplanin led to an increase, similar to saline solution. No significant side-to-side differences in average CSA were observed. Soaking in chlorhexidine decreased stiffness but increased stress at failure. None of the solutions affected load at failure.

## Funding

Funding for this study was provided by the National Institutes of Health (R01 AR071985).

## Supplementary Data

Appendix Fig 1 Setup of 3-dimensional scanning. **Appendix Fig 2.** The specimens were fixed via clamps during biomechanical tensile testing.
