# Supplementary material for: Soaking in Povidone-Iodine, Chlorhexidine, Teicoplanin, Vancomycin, and Saline Solution Differentially Alters Porcine Flexor Tendon Size and Biomechanical Properties
Source: Arthrosc Sports Med Rehabil. 2025 May 21;7(4):101168. doi: 10.1016/j.asmr.2025.101168 (PMC12447177; doi:10.1016/j.asmr.2025.101168)
Supplement: Appendix Fig 1 — Setup of 3-dimensional scanning. Appendix Fig 2. The specimens were fixed via clamps during biomechanical tensile testing. [file mmc1.docx]

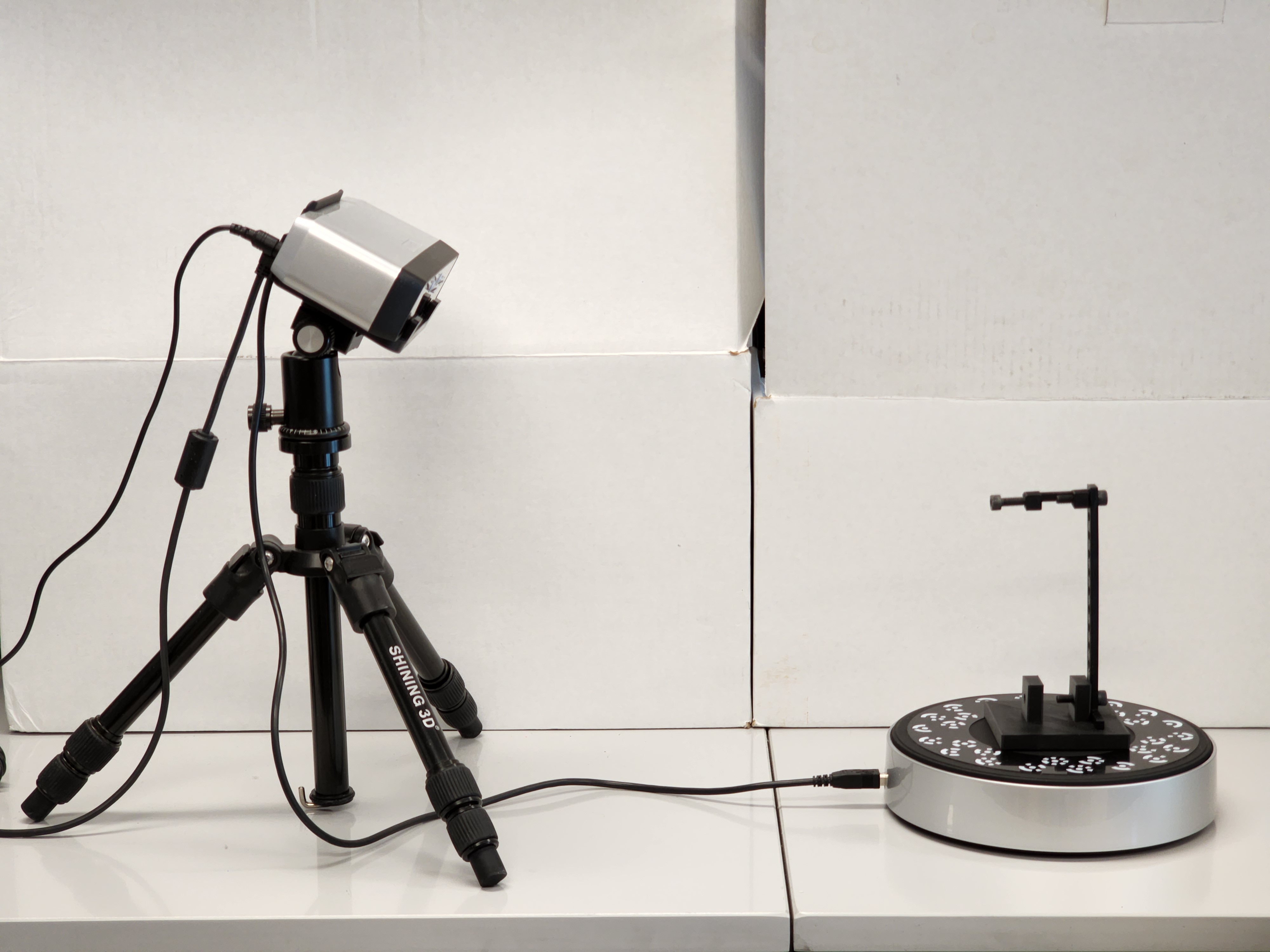


**Figure S1.** Setup of 3D scanning.


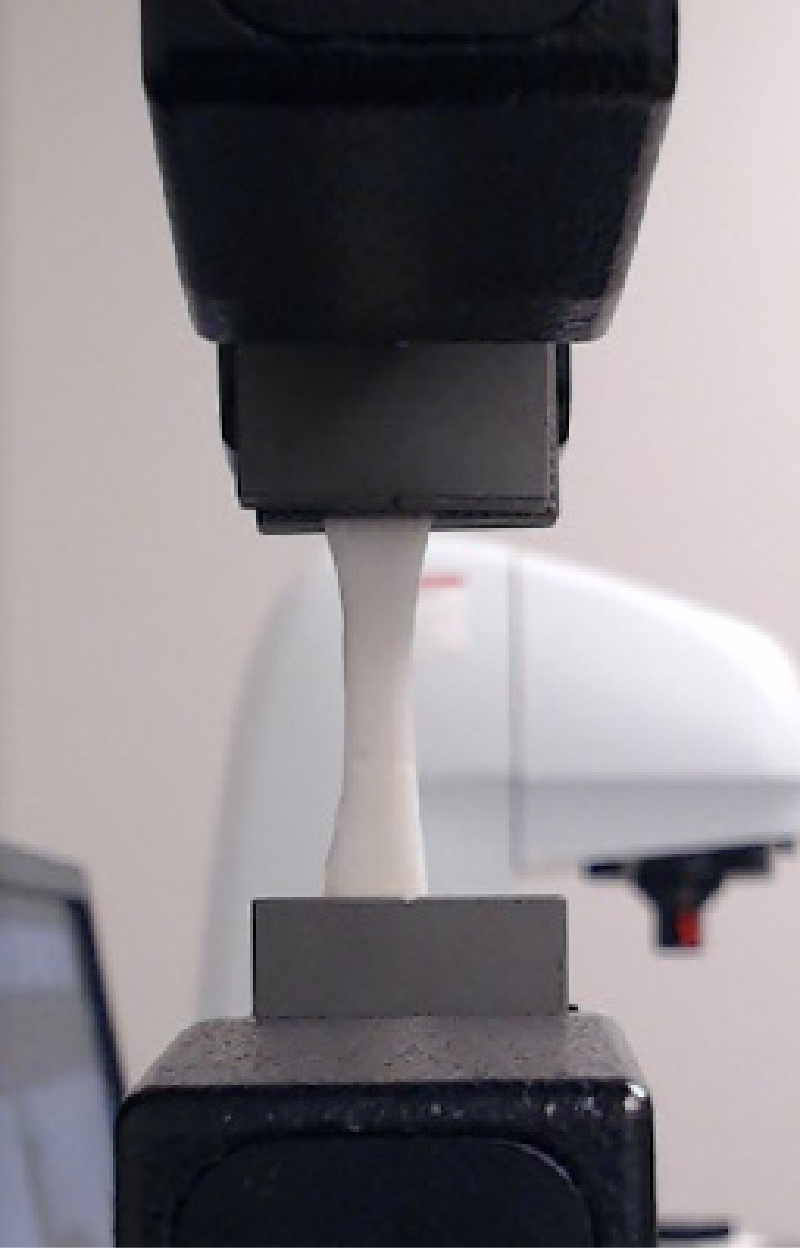


**Figure S2.** A specimen was fixed on clamps during the biomechanical tensile testing.
